# Conceptual-level workflow modeling of scientific experiments using NMR as a case study

**DOI:** 10.1186/1471-2105-8-31

**Published:** 2007-01-30

**Authors:** Kacy K Verdi, Heidi JC Ellis, Michael R Gryk

**Affiliations:** 1 Department of Computer Science, Rensselaer Polytechnic Institute, Troy, NY, 12180, USA; 2 Department of Computer Science, Trinity College, Hartford, CT, 06106, USA; 3 Department of Molecular, Microbial and Structural Biology, University of Connecticut Health Center, Farmington, CT, 06030, USA

## Abstract

**Background:**

Scientific workflows improve the process of scientific experiments by making computations explicit, underscoring data flow, and emphasizing the participation of humans in the process when intuition and human reasoning are required. Workflows for experiments also highlight transitions among experimental phases, allowing intermediate results to be verified and supporting the proper handling of semantic mismatches and different file formats among the various tools used in the scientific process. Thus, scientific workflows are important for the modeling and subsequent capture of bioinformatics-related data. While much research has been conducted on the implementation of scientific workflows, the initial process of actually designing and generating the workflow at the conceptual level has received little consideration.

**Results:**

We propose a structured process to capture scientific workflows at the conceptual level that allows workflows to be documented efficiently, results in concise models of the workflow and more-correct workflow implementations, and provides insight into the scientific process itself. The approach uses three modeling techniques to model the structural, data flow, and control flow aspects of the workflow. The domain of biomolecular structure determination using Nuclear Magnetic Resonance spectroscopy is used to demonstrate the process. Specifically, we show the application of the approach to capture the workflow for the process of conducting biomolecular analysis using Nuclear Magnetic Resonance (NMR) spectroscopy.

**Conclusion:**

Using the approach, we were able to accurately document, in a short amount of time, numerous steps in the process of conducting an experiment using NMR spectroscopy. The resulting models are correct and precise, as outside validation of the models identified only minor omissions in the models. In addition, the models provide an accurate visual description of the control flow for conducting biomolecular analysis using NMR spectroscopy experiment.

## Background

Scientific workflows are becoming much more widely used to concisely describe the activities required to execute scientific experiments [1-4]. While workflows found in the business world typically represent a consistent, repeatable sequence of events that operates on homogeneous data and conducts simple computations, scientific workflows represent a scientific experiment which can contain numerous unknowns, may have variability in execution sequence, operate on heterogeneous data, contain complex computations, and require a considerable degree of intuition on the part of the researcher to be executed successfully. Scientific workflows are complex, dynamic, and contain a high degree of variation.

The workflow for a scientific experiment can be modeled at two levels of abstraction; the implementation level and the conceptual level. Currently, most workflows created for scientific experiments are constructed using a scientific workflow environment such as Kepler [4]. Scientific workflow environments result in the creation of an implementation-level workflow, a workflow that represents how the system will carry out the tasks in the workflow. An implementation model of a workflow is tied to a specific set of tools to be used to carry out an experiment. In contrast, a conceptual model of a workflow focuses on the user's viewpoint without being tied to a particular set of underlying implementation tools. A conceptual model of a scientific workflow captures the researcher's understanding of what an experiment does and how it works without capturing implementation details. As such, a conceptual model highlights the data that flow through the workflow. The separation of the conceptual model from the implementation model allows the steps of the experiment process to be understood without constraints imposed by the implementation. In addition, once a conceptual model has been validated, it can be mapped to different implementations in different experiment environments.

The conceptual model describes what steps need to be taken in order to complete an experiment. For instance, in the analysis of biomolecules using NMR spectroscopy, one step in the conceptual model might be *Analyze Sample *which encompasses the tasks of *Construct Spectrum*, *Analyze Spectrum*, *Assign Peaks*, *Post Process Sample*, and *Validate Data/Results*. The implementation model describes the execution of the steps used to carry out the process described in the conceptual model. The implementation model describes the software, network access, database, and other resources needed to implement the workflow. For instance, the *Construct Spectrum *step of the conceptual model could be implemented using the NMRPipe tool [5].

Experienced scientists usually have a mental conceptual model of the workflow of an experiment. However, there is no widely acceptable method of codifying that model by writing it down in concrete format. Such an explicit conceptual model is essential for validating the correctness of the process of an experiment, and consequently ensuring the correctness of the experimental results. The conceptual model also serves numerous purposes from educating others about the process [6], sharing experimental procedures with other scientists, providing the opportunity to simulate new steps and processes, to streamlining experiments. The conceptual model can be used as the baseline for modifications to the system and for sharing tacit knowledge with other scientists. The development of this conceptual model is our current focus.

The process of conducting biomolecular analysis using Nuclear Magnetic Resonance (NMR) spectroscopy is a prime example of a scientific process which is complex, painstaking, time consuming, and requires a high degree of insight on the part of the researcher conducting the experiment. A typical experiment using NMR spectroscopy to determine the structure of a macromolecule involves the use of numerous stand-alone applications that use multiple different file formats requiring the NMR scientists to spend a significant amount of time formatting data for each application [7]. Due to the non-integrative nature of the scientific applications, most of the experimental data are fragmented and distributed across a variety of file formats risking the potential loss of valuable data. The variability of the NMR experimental process means that the detailed steps taken when conducting an experiment are rarely fully documented. This lack of documentation leads to difficulty in the reproducibility of experiments.

The approach to modeling the workflow for NMR experiments is part of a major research effort focused on the integration of numerous heterogeneous NMR applications [7,8]. The CONNecticut Joint University Research (CONNJUR) project is a multi-institutional effort investigating the construction of a framework to support the experimental process of employing NMR to determine protein structure. One of the goals of the CONNJUR project is to improve the efficiency of the experimental process and to provide data management support for that process by integrating existing tools and an underlying database to form an integrated environment to support NMR experiments.

### Scientific workflow systems

Due to the need for automating the execution of scientific experiments, numerous scientific workflow management systems have emerged in the academic community with leading systems including Kepler [4], Taverna [9], Triana [10], and WOODSS [3]. These scientific workflow management systems typically provide a distributed environment for scientists to conduct experiments by (remotely) connecting to existing collaborative scientific data sources to access and store relevant experiment data.

Kepler [1,4,11] is an open source scientific workflow management system designed to implement and execute scientific workflows in a distributed environment. Kepler uses Grid-based computing to access remote resources. Kepler uses actors to model the individual steps of an experiment at a fine-grained level of detail.

The WOrkflOw-based spatial Decision Support System (WOODSS) [3,12,13] was originally built as a workflow system and has been extended to support other scientific applications such as agro-environmental planning and bioinformatics. The main components of WOODSS are a Geographic Information System (GIS) and a Workflow Repository.

Specifically designed for the construction and execution of bioinformatics workflows, Taverna [9,14] is a scientific workflow management system that supports experiments which access numerous local and distributed information sources. A scientist constructs a scientific workflow at the functional level using the Simple Conceptual Unified Flow Language (Scufl).

Triana [10,15,16] is a workflow-based graphical problem-solving environment which was originally developed by scientists conducting the GEO600 gravitational wave experiment. Triana uses tasks (conceptually the same as actors in Kepler and processors in Taverna) to construct functional workflows.

These scientific workflow management systems all provide useful functionality for representing workflows at various levels of abstraction. However, none of these tools supports high-level conceptual modeling of the workflow for an entire scientific experiment nor do they provide guidance as to how to create such a conceptual model in the first place.

### Modeling notations for scientific workflows

A variety of modeling notations has been used to capture aspects of scientific workflows. Traditional computing notations have been used including UML, ontologies, Petri nets, and Data Flow diagrams. Notations constructed specifically for the scientific domain have also been created including an SQL-like language [17], the Abstract Grid Workflow Language (AGWL) [18], PLAN [2] and Virtual Data Language (VDL) [19]. We begin by discussing the use of traditional notations.

UML 2.0 is an object oriented modeling methodology commonly used for software engineering [20,21]. UML 2.0 Activity Diagrams have been utilized to model computational and business processes from an object-oriented perspective. Semantics used in Activity Diagrams are based on Petri Nets and are defined in terms of token flow [21]. Control flows and data flows can be represented with UML 2.0 Activity Diagrams.

Ontologies provide a description of a concept or domain, but do not model the active aspect of a workflow that shows how data move through a series of steps to accomplish a task. Ontologies provide a common naming and functionality convention for work-flow components used to construct scientific workflows. Ontologies are currently being explored as an approach to constructing workflows from disparate data or software components [22-24].

Petri nets have frequently been used to model the workflows of biological systems and medical processes [25,26]. Since Petri nets represent a view of the processes and flow of resources in a system, they are a natural modeling representation for scientific workflows.

Data Flow Diagrams have been used effectively to model how data move through the steps in a workflow. In one related work, an approach called Participatory Workflow Analysis [27] was used for domain analysis with a multi-disciplinary group of scientists to capture the different types of problems and solutions scientists encounter in their experiments for a problem solving environment. Data Flow Diagrams are used as the notation for the workflows.

The main advantage to using traditional notations for capturing scientific workflows is that the graphical notations are relatively easy for the lay person to understand. The drawback to such notations is that they frequently only represent the workflow from a single view point such as the flow of data through the workflow. In addition to traditional computing notations, a variety of Workflow Modeling Languages (WML)s has been developed to compose functional workflows for execution.

In a foundational work, Castro et al [28] define a comprehensive set of components and operators that are commonly used in scientific workflows. Built on this theoretical base, the GPIPE prototype is a workflow generator which allows users to create workflows consisting of these components and operators using a GUI.

A WML which is tightly integrated with SQL and uses SQL syntax has been developed as a framework for modeling and executing workflows [17]. Scientists construct the workflow by generating commands using the WML to create programs, inputs, and outputs.

The Abstract Grid Workflow Language (AGWL) [18] is an XML-based language that can be used to construct scientific workflow applications at the functional level of the workflow. PLAN [2] is a similar XML-based WML used to represent scientific workflows that analyze or search for data. Specifically designed for bioinformatics workflows, PLAN can be used to search and return results across numerous heterogeneous systems. Virtual Data Language (VDL) is a declarative language for modeling workflows. VDL uses a C-like language to represent the datasets and procedures of a workflow [19].

The main drawback to these WMLs is that they have a steeper learning curve than do most traditional computing notations. The WMLs require the user to learn some form of a text-based language. Workflow components are constructed using these work-flow modeling languages, which are then instantly executed to produce the results. In most instances, the languages do not support a graphical representation of the workflow itself.

While there are many widely accepted approaches for designing business workflows [29], the process by which scientific workflows are determined up to now has been ad hoc, or at least undocumented. We present a process for constructing workflows for scientific applications at the conceptual level.

## Results

Several factors motivated our selection of approach to modeling the workflow for biomolecular analysis using NMR spectroscopy. Our first goal was to construct a comprehensive and accurate conceptual model of the workflow, a broad model that encompasses the entire process, rather than looking in depth at any one phase of the NMR experiment process. A second goal was to manage complexity by providing different perspectives on the experiment process. The use of three phases of modeling supports discrete views of the experiment without loss of detail. Yet another goal was to use a modeling notation easily understandable to the NMR experimenter. In order to ensure the accuracy of the model, the model must be easily validated by the domain expert who may not clearly understand the intricacies of a complex text-based workflow modeling language. Similarly, we chose to use a component-based modeling approach that could represent scientific workflow components in a generic format. The use of a generic component-based modeling technique allows for ease of transformation to other modeling methodologies. Another factor that influenced our approach was time constraints. Since NMR researchers would typically prefer to spend their time conducting experiments, they have limited time to devote to modeling exercises. We needed to choose a proven, efficient and effective modeling approach for capturing the scientific workflows.

**Figure 1:**
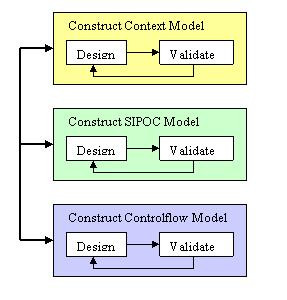
Conceptual Modeling Approach.

Due to the complex nature of the scientific domain, current state of implementation of the NMR applications, time constraints, and enormous data requirements, a top-down, iterative, design approach was chosen to capture the scientific workflows. The approach starts by looking at the highest level of the experiment and decomposing the steps in the experiment until a reasonable level of detail has been obtained. In order to capture the complexity of the experiment process, the approach includes three different models, as shown in Figure 1. The process consists of the following modeling phases: the context model which provides a structural view of the processes and their sub-processes; the Supplier-Input-Producer-Output-Consumer (SIPOC) model which highlights the data that are produced and consumed and how it flows during an experiment; and the control-flow model which demonstrates the flow and ordering of the steps in the experiment. Each phase consists of a two-step process of design and validation of the model. Each phase involves facilitated work-shops with subject matter experts (in our case the NMR spectroscopist). Since the three models provide three different perspectives on the workflow, the development or refinement of one model may result in the identification of missing elements in another modeling phase.

The process of constructing the conceptual level model of the scientific workflow for biomolecular analysis using NMR spectroscopy was performed collaboratively using facilitated workshops with three participants. One participant was an expert in business process modeling and served as the knowledge engineer, the second was a subject matter expert, in our case an expert NMR spectroscopist, and the third participant facilitated the meetings and documented the meeting proceedings. The NMR spectroscopist is a seasoned author on topics in the biomolecular analysis using NMR spectroscopy arena and has expertise in the area of Molecular, Microbial and Structural Biology. At the beginning of each workshop, models captured in the previous workshop were validated with our subject matter expert to ensure completeness before advancing to the next step in the modeling process.

A subset of the steps in the workflow for the process of conducting biomolecular analysis using NMR spectroscopy was captured. This subset was sufficient to completely exercise each of the three modeling levels and to express several processes at the finest level of granularity. Over the course of four months, we conducted eight workshops with a total duration of fifteen hours. Twenty four models were constructed during the workshops, using our process for conceptual-level workflow construction. We constructed the context model, all of the high-level SIPOC models for the top level processes, and constructed all but one of the scientific workflows for one of the more-complex sub-processes.

### The context model

The first step in the process of determining the NMR experiment workflow model is to create a context model. A context model is a proven tool used in system analysis to identify the scope and boundaries of a system being modeled, while setting the top level of abstraction for capturing the workflow [30]. A context model provides a structural perspective on the experiment process by describing the main processes and their sub-processes in a workflow. A single top-level context model is constructed for the system being modeled. Due to the complex nature of the experiment, we chose to focus only on the high-level processes in order to gain a common understanding of the scientific domain [6]. We modeled the experiment using a hierarchical view of the high-level processes. The hierarchical view of the high-level process is used to demonstrate the super-workflow (parent) and sub-workflow (child) relationship.

#### Context model components

**Figure 2:**
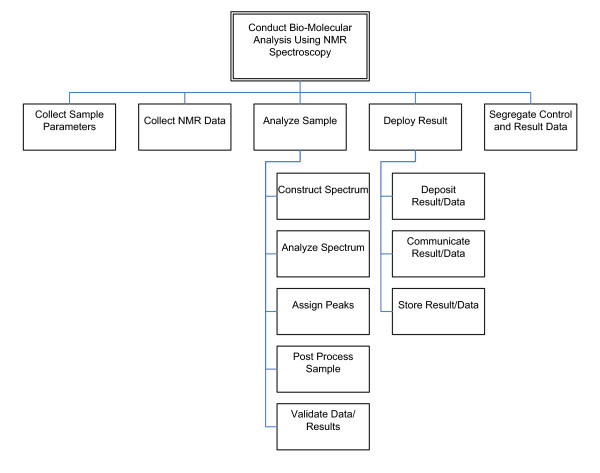
Context Model for the Process of Conducting Biomolecular Analysis Using NMR.

A context model consists of different kinds of a component called a process. A process in the context model represents a high-level activity that must be done to complete the experiment. The context model for the process of biomolecular analysis using NMR spectroscopy is shown in Figure 2.

The context model defines a hierarchy of processes that describes the workflow at the highest level with no explicit ordering. Processes are represented by rectangles. Only a single root process for each context model is allowed. The root process represents the main experiment being modeled and is typically located at the top of the diagram. The root process in our context model is *Conduct Biomolecular Analysis Using NMR Spectroscopy*. Top-level processes are those located directly below the root process such as *Collect Sample Parameters *and *Analyze Sample*. Each process that has processes connected below it is a parent or super process and the processes that are below the parent process are known as child or sub processes.

There are five separate sub-processes to *Analyze Sample*. *Construct Spectrum *is the process of converting NMR data describing the bulk magnetic oscillations of the sample to a spectral frequency map of those oscillations. *Analyze Spectrum *is the process of identifying the frequencies at which oscillations occur. *Assign Peaks *is the process of identifying which nuclei in the sample are responsible for the observed signals. *Post Process Sample *encompasses a whole host of various processes which are workflow dependent, such as the identification of components of biomolecular secondary structure. *Validate Data/Results *is the process of confirming that the processed data are accurate.

A two-hour workshop was conducted to capture the context model. An initial process model [7] demonstrating the current state of NMR data analysis was used as the starting point. An initial context model containing the main process of *Conduct Biomolecular Analysis Using NMR Spectroscopy *and its five sub-processes was constructed. The *Analyze Sample *and *Deploy Result *processes were then further decomposed due to the complexity of these two processes. The context model was considered to be complete once the domain expert indicated that all high-level processes had been identified. Verification of the completeness was performed via a walk-through of the context model that verified that every step in the experiment was accounted for at the highest level of abstraction. At the conclusion of the workshop, the context model was distributed to all workshop participants who performed a final, independent validation. Minor changes were incorporated into the model at the beginning of the subsequent meeting.

The context model identified the areas of the workflow that required further analysis in subsequent workshops to capture the SIPOC models and workflow models. The context model also helped us make judgments about whether a process/activity was out of scope for the process of conducting biomolecular analysis using NMR spectroscopy.

### The SIPOC model

Once a complete context model is constructed and modeling participants have gained an understanding of the major processes and their decompositions, the second phase of modeling may begin. In the second phase of modeling, the processes identified in the context model are further detailed with corresponding producers, data inputs and outputs, and consumers using a SIPOC model. The SIPOC model is based on the Supplier-Producer-Customer (SPC) chain, a proven modeling technique used in Continuous Process Improvement [31]. The SIPOC model was chosen as it allows both data and process flows to be captured (at a high level) using a single diagram notation. While modeling the scientific workflows, we retained the label "consumer" for the Customer link in the SPC chain as output in a scientific experiment is typically consumed by a connecting process or entity. SIPOC modeling is performed for all processes identified in the context model. Any complex processes identified during SIPOC modeling may be further refined by applying SIPOC modeling to those processes. The goal of the SIPOC phase of modeling is to iteratively apply the SIPOC model to various processes until a model that is sufficiently low-level as to be modeled using a flow chart is obtained. Only the top level of the context model is always included in the SIPOC model. The decision as to whether to model lower levels of the context model in the SIPOC model is based on the complexity of those levels of the context model.

**Figure 3:**
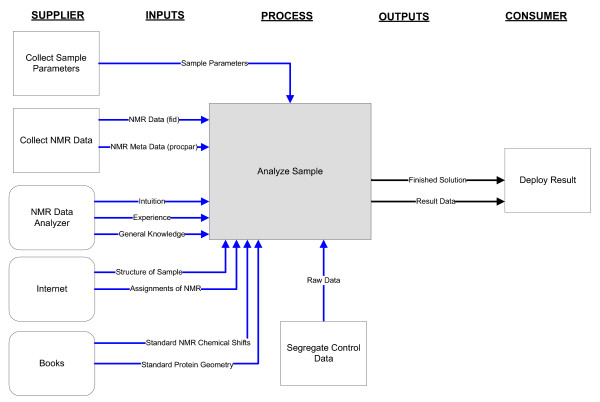
SIPOC Model for the Analyze Sample Top-Level Process of the Process of Conducting Biomolecular Analysis Using NMR.

We decided to model a high-level view of the five top level processes identified in the context model. Figure 3 shows the SIPOC model for the *Analyze Sample *top-level process identified in the context modeling phase.

As shown in Figure 3, the top level process, *Analyze Sample*, collects input data from many types of suppliers. Three of the suppliers are other processes which occur elsewhere in the work-flow: *Collect Sample Parameters*, *Collect NMR Data *and *Segregate Control Data*. Other input comes from human sources: the *NMR Data Analyzer *process, as well as information deposited in reference literature and the internet. All of this data is required by the *Analyze Sample *process to produce a final result, which is composed of both the unrefined, resultant data as well as the refined solution for publication and deposition.

#### SIPOC model components

The SIPOC model uses five different kinds of components:

(1) Supplier – an entity that supplies the input to the process. Suppliers may be other entities, represented by rectangles or rounded rectangles with arrows flowing outwards towards the process to which the supplier supplies input. The *Collect Sample Parameters *process is a supplier to the *Analyze Sample *process shown in Figure 3.

(2) Input – any information, data, or objects supplied to the process, represented by arrows flowing in the direction towards the *Analyze Sample *process. *Sample Parameters*, *NMR Data*, and *Standard NMR Chemical Shifts *are inputs consumed by the *Analyze Sample *process shown in Figure 3.

(3) Process – an activity required to transform an input to an output, represented by a rectangle with arrows flowing into and out of the rectangle. In Figure 3, the *Analyze Sample *process consumes input from the *Collect NMR Data *process, among others, and produces output to be consumed by the *Deploy Result *process.

(4) Output – any information, data, or object created by the process, represented by arrows flowing out of the main process being modeled. In Figure 3, the *Analyze Sample *process has two outputs, *Finish Solution *and *Result Data *which serve as input to the consumer *Deploy Result *process.

(5) Consumer – an entity that uses the output from a process, represented by a rectangle with arrows flowing into the rectangle. In the *Analyze Sample *process shown in Figure 3, the *Deploy Result *process consumes the *Finished Solution *and *Result Data *outputs from the *Analyze Sample *process.

SIPOC models were constructed for five top-level processes identified in the context model (see Figure 2). The process used to capture the SIPOC models for the process of biomolecular analysis using NMR spectroscopy involved three two-hour workshops and one one-hour workshop with workshop participants performing independent reviews of the models produced in a workshop in-between meetings. Validation of a SIPOC model consisted of verifying that all producer, inputs, outputs, and consumers were captured correctly in the model.

In order to demonstrate the iterative application of the SIPOC model, the *Analyze Sample *SIPOC model was further decomposed into a more-detailed SIPOC model. One two-hour workshop and one one-hour validation workshop were conducted to capture the *Analyze Sample-Level 2 *SIPOC model. The first workshop began with a validation of the five higher level SIPOC models created in the previous workshop. To gain an understanding of the *Analyze Sample *process, we constructed a context model for the *Analyze Sample *process. We used the context model as a tool to identify all the major sub-processes that compose the *Analyze Sample *process. Our context model identified the *Construct Spectrum *sub-process as needing further decomposition.

The *Analyze Sample *context model and *Analyze Sample – Level 2 *SIPOC model were validated for completeness during the one-hour validation workshop. Validation consisted of a walkthrough that ensured that all supplier and consumer inputs/outputs had been identified for the process. In addition, the model was checked to ensure that all parent SIPOC components were captured within all the sub-process SIPOCs.

The SIPOC models proved to be a useful visual tool for providing the team with a common understanding of the process of conducting biomolecular analysis using NMR spectroscopy. Once the *Analyze Sample – Level 2 *SIPOC model was validated, we proceeded to the next phase in the process to construct the control flows.

### The control-flow model

Once the major processes of the conceptual model of the workflow have been identified and described using the SIPOC model, the third phase in our process is capturing the flow of control of the scientific workflow. We chose to use a notation that closely resembles flow charts to represent our scientific workflows as flow charts are a proven modeling technique used to accurately capture the control flow of processes. In addition, flow chart modeling is a simple and efficient method to identify the activities required to execute a process [32]. Decomposition of flow charts is possible if the primary process does not provide sufficient detail for the analysis of the process. The lowest decomposition we needed to capture for our conceptual-level view of the process was four levels of decomposition.

**Figure 4:**
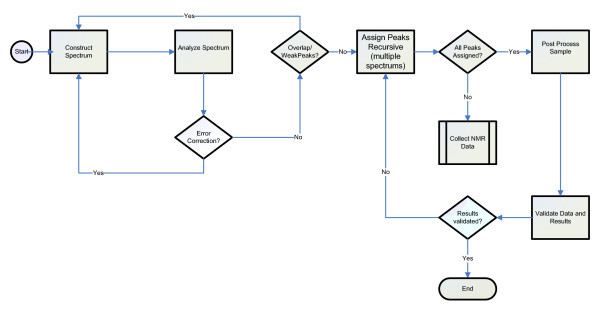
Control Flow Model for the Analyze Sample Process of the Process of Conducting Biomolecular Analysis Using NMR.

The *Analyze Sample *process was chosen for decomposition as it includes a significant portion of the complex computations and analysis found in the process of biomolecular structure determination and is a process that would highly benefit from automation. Figure 4 shows the control flow for the *Analyze Sample *process.

The *Analyze Sample *process control flow illustrates the iterative decision making characteristic of scientific workflows. The flow starts with a reconstructed spectrum to be analyzed. The spectrum is analyzed for both accuracy (error correction) and quality (overlap/weak peaks). Once the spectrum meets both requirements, the observed peaks are assigned iteratively, often requiring tentative assignments to be fed to later stages of the flow to be confirmed/denied, or even to retreat to prior stages of the flow and collect more data. Once all peaks have been assigned, and all post-processing results validated, the control flow ends.

Control flow modeling uses the following components:

(1) Start – a circle is used to represent the starting point of the process.

(2) End – an oval is used to indicate the termination point of the process.

(3) Activity – a task or step required to execute the process, represented by a plain rectangle. Some examples of activities used in our workflows are *Construct Spectrum *and *Analyze Spectrum*.

(4) External Process – a process that is external to the workflow which indicates the control flow of the workflow exits to an external process. Represented by a double barred rectangle, one example of an external process shown in Figure 4 is *Collect NMR Data*.

(5) Flow – the sequencing order of the activities of the process, represented by a directed arrow. An example of flows used in our workflows is the connection between the *Construct Spectrum *and *Analyze Spectrum *activities.

(6) Decision – a decision point in the flow of control where control could go one of two directions, represented by a diamond. The decision identifies the direction in which the flow moves based on the answer to a true/false question. For example, peaks are assigned until the results are validated as shown by the *Results validated? *decision.

Three two-hour workshops were conducted to capture the control flow for the *Analyze Sample *process. Since the validation for the *Analyze Sample *context model and *Analyze Sample Level 2 *SIPOC models was already complete, we immediately started constructing the control flow for *Analyze Sample *based on the sub-processes identified in the *Analyze Sample *context model.

In addition, the control flows for the *Construct Spectrum*, *Analyze Spectrum*, and *Post Process Sample *processes were captured in the first of the three workshops using the same process we used to construct the *Analyze Sample *context model and control flow. Validation of the *Analyze Sample*, *Construct Spectrum*, *Analyze Spectrum*, and *Post Process Sample *control flows were conducted at the beginning of the second workshop. The *Analyze Sample – Level 2 *SIPOC model and the process context models served as excellent references to verify that correct information was being captured in the control flows. By studying the inputs and outputs identified in the *Analyze Sample Level 2 *SIPOC model, the NMR domain expert was able to identify potential gaps in the lower level control flows. Context models were used to verify that all processes were represented in the lower level workflows. The context models served as a tool for determining which sub-processes needed to be decomposed. Control flows for seven additional processes were modeled and validated in the two last workshops.

### Validation of approach

In order to determine the soundness of the approach used to construct conceptual-level workflows for the analysis of biomolecules using NMR spectroscopy, we validated the models produced and the approach used with an outside, highly respected NMR spectroscopist. The validation approach was based on best practices used in systems analysis and design, software engineering, and business process modeling. Due to the size of the problem space and the number of models produced, we identified a subset of the models to use during validation of our approach. The *Construct Spectrum *process (see Figure 2) was chosen because it is the first step in *Analyze Sample *parent process and because its sub-processes decompose to a relatively detailed level.

The validation approach involved three steps. First, criteria for validation of the models were developed in the form of a checklist. Criteria were chosen in the hopes of identifying potential gaps or errors in the process. The spectroscopist responsible for validation was provided all checklists and we solicited feedback from the spectroscopist to ensure our criteria were complete. Second, the documents were provided to the reviewer for independent review. Lastly, one three-hour workshop was conducted where the spectroscopist responsible for validation verbally walked through all of the models for the *Construct Spectrum *process, checking for completeness and correctness of the models.

Validation proceeded in a top-down manner as the workshop began with a brief introduction to the *Construct Spectrum *context model. The context model was validated using the validation criteria checklist. The SIPOC modeling constructs were explained to the spectroscopist responsible for validation and the SIPOC models for the *Construct Spectrum *process were validated. Lastly, the control flows for the *Analyze Sample*, *Construct Spectrum*, *Convert Data from Time Domain to Frequency Domain*, *Set Order for Execution of Functions*, and *Configure Values for Functions *processes were validated using the validation criteria. The ease of understanding our modeling approach was highlighted when, during the review of the control flows, the spectroscopist responsible for validation spontaneously began constructing a flow chart to represent an additional decision branch for the *Convert Time Domain to Frequency Domain *control flow.

Only minor issues were identified during the validation workshop. For example, a process name was changed to more accurately describe the functionality provided by the process, the outputs and consumer on the *Analyze Sample *SIPOC model. The control flow of the *Analyze Sample *process was slightly modified to include an additional decision. The NMR spectroscopists agreed that our models served as an accurate representation of the conducting biomolecular analysis using NMR spectroscopy experiment. The full documentation of validated workflows is available at [33].

## Discussion

At the beginning of the procedure to determine the conceptual workflows for conducting an experiment to determine the structure of a molecule using NMR spectroscopy, the NMR spectroscopist expressed doubt that it would be possible to capture the steps of the experiment in an accurate and complete manner. This doubt arose from an understanding of the complexity of the experiment process, the variability that may occur during an experiment and the intuition required on the part of the scientist conducting the experiment. However, the clarity, correctness, and completeness of the models produced using our approach clearly indicated that the approach was a success.

Nonetheless, there were several factors that limited the process developed to capture scientific workflows at the conceptual level. First, the complexity of the process of biomolecular analysis using NMR spectroscopy required that a simple modeling approach be used. An uncomplicated approach and correspondingly straightforward notation are necessary for all participants in the process to understand the nature and details of the workflow steps.

The limited number of NMR scientists imposed another restriction on the development of our approach. Based on the number of NMR scientists available in our area, our user base for capturing the workflows was limited to one expert spectroscopist with a second NMR spectroscopist available for validation purposes. Because of the NMR scientist's credentials and maturity in the field, we believe we were able to construct a set of reasonable and implementable scientific workflows for the process of conducting biomolecular analysis using NMR spectroscopy.

Overall, the results of our process to construct conceptual-level workflows for the experiment of conducting biomolecular analysis using NMR spectroscopy appear to be correct and precise. However, they are not necessarily unique. In analogy to the association rules of addition, there may be many alternate paths to the same correct conclusion. Such prejudices of researchers towards pet methodology must be addressed during the validation phase. Codifying scientific processes in terms of workflows ultimately allows scientists to share, compare and critique their individual, disparate solutions to common problems. We were able to document numerous processes accurately in a short amount of time. The iterative nature of the process gave us the flexibility to re-use modeling techniques from previous phases, which reduced the time required to capture the models. During the validation of the process, we encountered only minor issues with the models. The NMR spectroscopist responsible for validation agreed that the models provide an accurate visual description of the control flow for conducting biomolecular analysis using NMR spectroscopy experiment. We did observe that when workshops were conducted on a regular basis (e.g., weekly), the modeling process flowed more smoothly and was more productive, resulting in a greater number of models generated per meeting. For instance, when workshops were held on a weekly basis, we were able to construct twice the number of models than when workshops were held less frequently, since the methodology and models were fresh in our minds. We recommend identifying a subset of models to capture for a process and then, if possible, schedule the meetings at least once a week until the subset of models is constructed.

### Benefits of the approach

The process of capturing and designing the workflows for the analysis of biomolecules using NMR spectroscopy highlighted several resulting benefits. The modeling process itself caused the NMR spectroscopist to gain a more-comprehensive understanding of the steps of the experiment process and their relationships to one another. The process of determining the context, SIPOC, and control flow models permitted the spectroscopist to focus on the experiment process at various levels of abstraction and from various viewpoints, allowing the spectroscopist to gain new understanding of how the steps in the experiment are related. This understanding has the potential to lead to new and hopefully more-efficient approaches to conducting experiments.

The NMR spectroscopist also benefits from the codification of the steps taken to conduct an NMR experiment. Currently much of the information about how to construct an experiment for determining the structure of macromolecules using NMR spectroscopy is transferred among spectroscopists verbally or with minimal text in an informal manner. The set of models created can serve as common foundation for spectroscopists to discuss, compare and evaluate their experiment process, as well as serving as a base for teaching new spectroscopists how to conduct an experiment.

Codifying the workflow also provides a conceptual foundation for modeling the intermediate, derived data which are produced during biomolecular analysis. Having such a data model in place is critical for the aforementioned goal of CONNJUR, to provide an integrated desktop environment for biomolecular NMR data processing. This, in turn, will allow the workflow to be expressed intuitively in the design of the integration application and thereby allowing the application to convey the experimental methodology to the novice spectroscopist.

The ease with which the models are understood makes the approach straightforward to learn and use by NMR spectroscopists. In the case of the validating spectroscopist, that spectroscopist started constructing control flow models with only forty-five minutes exposure to the overall approach. In addition, the minimal amount of rework required for the validated models demonstrates that our models accurately represent the conducting biomolecular analysis using NMR spectroscopy experiment.

The simplicity of the models and the iterative approach that focuses on control flow used in our approach allows precise models to be constructed relatively quickly. In comparison to a similar effort, Participatory Workflow Analysis [27], our models contain more detail about the experiment and provide a high-level view of the entire process, including activities, inputs, outputs, suppliers and consumers.

## Conclusion

The approach to the creation of conceptual scientific workflows described in this paper uses a broad model intended to encompass the entire experiment process. The goal is to accurately model the workflow of an NMR experiment which contains the complexity representative of other scientific experiments. Based on our experience with modeling the intricate experiment of biomolecular analysis using NMR spectroscopy, we believe that our approach can be used by the broad audience of the general scientific community.

Our future work in the area of conceptual modeling of scientific workflows has two main directions. One topic for further investigation is the implementation of the existing workflow models for the process of conducting biomolecular analysis. This implementation could be carried out using a scientific workflow management system, such as Kepler [4], Taverna [9] or Triana [10]. Once a system is chosen, conceptual models would be converted into corresponding implementation models, starting with the decomposed *Analyze Sample *process. UML 2.0 Activity Diagrams would allow the modeling of both the control flow and data flow in one model diagram and UML is the de facto industry standard for software requirements. In addition, conceptual-level workflows can easily be converted to UML 2.0 notation.

A second area of investigation is the application of the process to the modeling of scientific workflows in other domains. The application of our process to domains such as biochemistry, chemistry, and biology would demonstrate the utility of our approach across a range of scientific workflows.

Additional areas of investigation would be workflow sharing, collaboration, connectivity with instruments and data stores and the design of user interfaces. The use of the workflow for aiding students learning the NMR experiment process is also being explored.

## Methods

The method used to capture the workflow for conducting an NMR experiment is an iterative, top down, approach that starts by looking at the experiment from the highest level of abstraction and decomposing the steps in the experiment until a reasonable level of detail has been obtained to represent the experiment. Our process consists of three different modeling phases: context model, SIPOC, and control flow. Each phase provides a distinct view of the workflow and consists of a design phase and a validation phase to ensure the correctness and completeness of the models constructed. Each phase involves the use of facilitated workshops with subject matter experts (in our case the NMR spectroscopist). The models from each of the phases were captured using Microsoft Visio.

The process begins by constructing a context model of the NMR experimentation process. The second phase of the process involves capturing SIPOC models for all the high level processes identified by the context model. The SIPOC models provide a detailed overview of each high level process. The third phase of the process revolves around capturing the workflow using a flow-chart-like notation. Workflows are decomposed into sub levels until the appropriate level of detail is represented in the workflow.

## Abbreviations

NMR – Nuclear Magnetic Resonance

## Authors' contributions

KKV was responsible for conceptualization and design, took part in implementation, and participated in writing the manuscript, HJCE took part in implementation and wrote the first draft of the manuscript, MRG was the NMR domain expert, took part in implementation and participated in writing the manuscript.
